# Students’ awareness of the bruxism causes, effects and therapies

**DOI:** 10.1016/j.heliyon.2023.e23708

**Published:** 2023-12-19

**Authors:** Mateusz Gizler, Natalia Pietrzak, Klara Saczuk, Monika Lukomska-Szymanska, Barbara Lapinska

**Affiliations:** a Faculty of Dentistry, Medical University of Lodz, 251 Pomorska St, 92-213 Lodz, Poland; b Department of General Dentistry, Medical University of Lodz, 251 Pomorska St, 92-213 Lodz, Poland

**Keywords:** Awareness, Knowledge, Bruxism, Temporomandibular disorders, Survey, Students

## Abstract

**Objectives:**

Bruxism is a repetitive activity of the masticatory muscles characterized by clenching or grinding teeth and/or mandibular stiffening. Bruxism manifests itself in two forms: during sleeping and waking. The etiology of bruxism is multifactorial. The treatment of bruxism is mainly based on making the patient aware of the presence of the condition. The aim of the study was to assess knowledge on the causes and the effects of bruxism among Polish students as well as the possible management.

**Materials and methods:**

The anonymous online survey was conducted among students (aged between 18 and 25 years old) of universities across Poland, using the Google Forms platform. The survey contained questions concerning bruxism causes, effects and therapies. All variants of answers in these questions contained true information about bruxism.

**Results:**

The study found significant differences in awareness of bruxism among genders in favor of females. The awareness of bruxism among population residing in cities and in villages was comparable (*p* > 0.05). However, the results should be taken with care due to limited number of students that participated in the study and uneven gender distribution among urban and village residents.

**Conclusions:**

Within the limitations of the study it can be concluded that the knowledge of bruxism among Polish students is higher in comparison with the findings from the literature, as well as the superiority of women's awareness of bruxism over men. Future studies should be conducted on greater student population, with even distribution of participants among country areas and variety of universities.

**Significance:**

The findings may indicate the need for further education of male young adults, on bruxism causes, possible effects and therapies to increase their awareness of bruxism and encourage early diagnosis and treatment.

## 1 Introduction

Bruxism is a common problem in modern society. Previous studies showed that the occurrence of bruxism ranges between 5.9 % and 49.6 % in children [1] and between 26 % and 66 % in adult patients with temporomandibular disorders (TMDs) [2].

The word “bruxism” comes from the Greek word “brugmos”, meaning “gnashing of teeth” and is characterized by grinding and clenching of the teeth and/or by bracing or thrusting of the mandible [[3], [4], [5]]. In 2018, Lobbezoo et al. [6], during the International Consensus on Bruxism, reformulated the 2013 definition of bruxism as a repetitive activity of the masticatory muscles during sleep characterized as rhythmic (phasic) or non-rhythmic (tonic) and referred to as sleep bruxism, and during wakefulness - characterized by repetitive or sustained tooth contact and/or by bracing or thrusting of the mandible and referred to as awake bruxism.

Bruxism should not be considered as a disorder in otherwise healthy people, but rather as a behavior that may be a risk factor for the development of clinical consequences [6]. Bruxism is considered to have the most destructive and harmful effect on the orofacial area [7]. The etiopathogenesis of bruxism has not yet been clarified. Receptors that are sensitive to stretching and increasing muscle tone are neuromuscular spindles. Their change in excitability is the basis of the mechanism of bruxism formation and it persists even after cessation of motor activity. This leads to ischemia of the tissue, resulting in pain [8].

Among the causes of bruxism, peripheral and central factors are mentioned [3]. Peripheral factors include psychological factors (stress, anxiety, personality components) and genetic factors [8]. Central factors include disturbances at the level of the neurotransmitters and the basal ganglia, as well as organic and functional changes of the nervous system [8]. The organic causes of bruxism are: craniofacial injuries, tetanus, epilepsy, cerebellar hemorrhage, meningitis, dysfunction of dopaminergic transmission in Parkinson's disease and genetic factors [3,8].

Additionally, other causes of bruxism are psychosocial factors like stress and pathophysiological factors (e.g. illness, trauma, genetics, smoking, caffeine, medication or drugs intake), sleep disorders and involving dopaminergic system [9].

Among non-occlusal causes of bruxism habitual gum chewing stands out, followed by nail and/or skin biting around nails, lip, cheek and biting objects [10]. Other causes of bruxism involve occlusal factors and many types of dental interventions (poorly fitting fillings or dentures), including orthodontic treatment [11]. The majority of orthodontists believe that orthodontic intervention is not the best option for TMD treatment, whereas it can induce TMD symptoms [12]. For many years, bruxism was being connected with malocclusion and anatomical anomalies [13,14]. Due to the lack of adequate clinical studies, this relationship has been insufficiently investigated and documented [15].

An additional predisposition for bruxism is chronic stress, which leads to emotional tension and thus to increased muscle activity [10]. Studies have found the relationship between perceived stress and bruxism symptoms [16,17]. Prolonged muscle contraction by the limbic system causes damage to the stomatognathic system. It has also been proven that stress-induced bruxism occurs more frequently in women [8,18,19].

When the teeth are clenched, the contraction of masticatory muscles occurs, which leads to an increased tension in the centric occlusion. Increased muscle activity increases the risk of negative effects on oral health, e.g. pain in the masticatory muscles or temporomandibular joints (TMJ), tooth abrasion [6,15,20]. Increased tension may also lead to hypertrophy of the masticatory muscles, especially the masseter muscle. This symptom is called “square face” [7]. Masticatory muscle pain can disrupt everyday functions such as sleep, appetite, swallowing and speaking [7]. According to the analysis of Ciancaglini et al. [21], bruxism is also one of the causes of disorders in the temporomandibular joint (TMJ) and is significantly associated with craniofacial pain, difficulties in closing or opening the mouth, TMJ sounds and neck pain. Bruxism is also characterized by a destructive effect on teeth, resulting in tooth wear and/or loss of dental fillings and prosthetic crowns. Pain, pathological mobility recession, bleeding gums and even fracture of teeth can be observed [3].

In addition to deteriorating the quality of life of a person with bruxism, it can also have negative impact on relationships with a partner. The clenching and grinding of teeth in bruxism during sleep may impair the sleep quality of the partner [3,22].

Due to the multifactorial etiology of bruxism, is not possible to completely eliminate this problem. However, there are methods that can significantly improve the patient's quality of life mainly by reducing the muscle activity and managing pain. Psychological counselling, occlusal stabilizing splint or pharmacological therapies are often used to manage bruxism. Introducing lifestyle changes, reducing stress, counselling or hypnotherapy are major components of bruxism management [23]. Properly done orthodontic treatment can reduce the likelihood of subsequently developing TMD [11]. One of the methods of reducing the symptoms of bruxism is the use of the botulinum toxin, which reduces the nerve conduction thus reducing the activity of the masticatory muscles [24]. Pharmacological agents such as clonazepam and l-dopa are also used to reduce the symptoms associated with bruxism. They significantly improve the quality of sleep and reduce the incidence of bruxism episodes. On the other hand, there are also pharmaceuticals that aggravate the symptoms of bruxism and worsen the quality of sleep, mainly haloperidol and propranolol [25].

In case of awake bruxism, treatment is based on making the patient aware of the presence of parafunction and the need to control excessive muscle tension and clenching of teeth. The use of biofeedback and relaxation techniques has positive effects [26].

Managing sleep bruxism consists of the application of bite splints. which contribute to tissue protection, relaxation of the masticatory muscles and relief of the TMJ. Attention should be also paid to improve the patient's sleep quality and to eliminate muscle pain. For this purpose – occlusal, pharmacological and behavioral therapies are used [27].

The main reason for conducting the following research is the increasing phenomenon of bruxism among students. This may be due to the long-term stress associated with studying and the exam session. In addition, the period of the COVID-19 pandemic and the related stress resulting from lockdown and fear of the unknown could have been the factor that would aggravate the above problem [17]. Students were selected as the target group of respondents due to their increased exposure to stress. In addition, while in lockdown, it was impossible to conduct personal surveys or clinical examinations, hence conducting an online survey was the most efficient way to reach the student respondents. Moreover, to our knowledge there has been no similar research on bruxism awareness conducted in population of students, especially during the COVID-19 pandemic.

The aim of the study was to assess the awareness of bruxism among Polish students, to estimate their knowledge on causes, effects and management of bruxism. With this study we wanted to increase awareness of bruxism symptoms among young adults that would contribute to faster diagnosis and seeking professional dental help. Introducing therapeutic measures for bruxism at early stage leads to decrease in complications related to bruxism. We hypothesized that: (1) women are more aware of the causes, treatment methods and effects of bruxism than men; (2) residents of urban areas have greater awareness of bruxism than the village population.

## 2 Materials and Methods

### 2.1 Study design

An anonymous online survey was carried out among students (aged between 18 and 25 years old) from Polish universities across the country, using the Google Forms platform (Appendix A). The questionnaire was sent to various universities, including medical and technical ones. Participation in the study was voluntary. The conduct of the reassert in the form of a survey lasted 5 working days from January 19, 2021 till January 25, 2021.

The institutional Research Ethics Committee approved the investigation (RNN/111/22/KE), and it was conducted according to the principles expressed in the Helsinki Declaration. The students received written information on the purpose and procedures of the study, and they gave written informed consent.

The survey included a total of 9 multiple choice questions, of which the first four concerned the persons details of the respondents (gender, marital status, place of residence and voivodship). Another 5 questions were directly related to bruxism (its causes, effects and treatment methods). All variants of answers in these questions contained true information about bruxism. The respondent also had the option “I don't know” to choose from.

The awareness of bruxism's (1) causes, (2) effects and (3) management therapies was estimated based on the number of correct answers selected by the respondents in each of the questions from 6 to 10 as follows.

1) awareness of bruxism causes was considered if chosen ≥60 % of the correct answers in question #6;
2) awareness of bruxism effects
a) on masticatory muscles and TMJ if chosen ≥60 % of the correct answers in questions #7;
b) on teeth and gums if chosen ≥60 % of the correct answers in questions #8;
c) other effects if chosen ≥60 % of the correct answers in question #9;
d) the overall awareness of the effects of bruxism was calculated based on the overall number of correct answers chosen in questions 7–9: when ≥60 % of correct answers were chosen, it was considered as being generally aware of the effects of bruxism
3) awareness of the bruxism management therapies if chosen ≥60 % of the correct answers in question #10.

### 2.2 Statistical analysis

Statistical analysis was performed using Statistica (Statsoft, Cracow, Poland) and RStudio as an environment for the R language.

In order to conduct a statistical analysis of the results obtained in the study, the frequencies of correct answers given by men/women and city/village residents were presented. The relationships between the choice of correct answers and the gender of the respondents, as well as between the choice of correct answers and the place of residence, were analyzed using Pearson's chi square test of independence.

Dependencies for which the calculated test value turned out to be equal to or greater than the critical value according to the chi-squared distribution tables for the appropriate number of degrees of freedom and the error probability *p ≤* 0.05 were considered statistically significant.

## 3 Results

### 3.1 Demographic characteristics of respondents

Of the four hundred and nineteen respondents, aged 18–25, women constituted 78.0 % (327) of all respondents, while men were 22.0 % (92) of all respondents. In addition. none of the respondents indicated an undetermined gender.

The vast majority (95.0 %, n = 398) of respondents were single (bachelor/maiden). People, who had a different marital status, e. g. divorced or those who refused to declare their marital status, accounted for 4.5 % (19) of the respondents. Only 0.5 % of respondents were married.

**Fig. 1:**
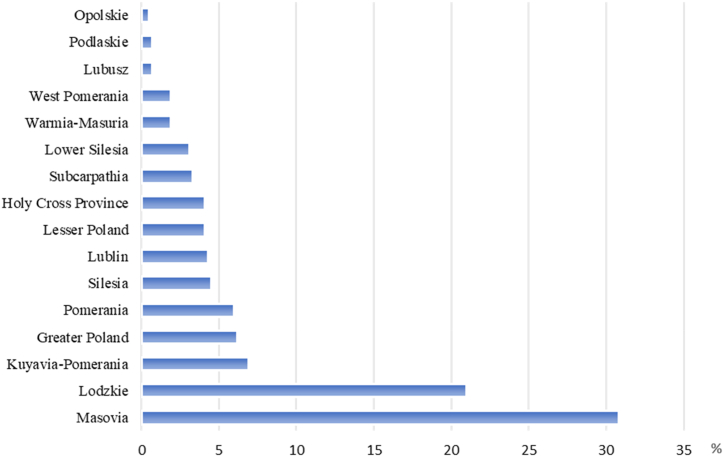
Responders in [%] by voivodeship.

The city population accounted for 69.7 % (292) of all respondents, while the village population was 30.3 % (127). The surveys were conducted in 16 voivodeships of Poland, with the largest number of respondents living/studying in the Masovian (30.8 %) and Lodzkie (21.0 %), while the lowest number of respondents were reported in the voivodeships of Lubusz (0.7 %), Podlaskie (0.7 %), and Opolskie Voivodeship (0.5 %) (Fig. 1).

### 3.2 Knowledge of the causes of bruxism

Among the causes of bruxism, the respondents most often depicted chronic stress (82.6 %) (Table 1), followed by mental disorders (74.9 %) and malocclusion (59.9 %). While the rarest cause of bruxism acknowledged by the respondents were genetic factors (22.9 %).

**Table 1:** Distribution of the listed causes of bruxism among all respondents.

Causes of bruxism	n	%
Chronic stress	346	82.6
Mental disorders, e.g. neurosis, depression	314	74.9
Malocclusion	251	59.9
Poorly fitted dentures or poorly made fillings	155	37.0
Frequent chewing gum and/or biting nails	138	32.9
Genetic factors	96	22.9
I don't know	34	8.1

Both, women and men most often chose prolonged stress, mental disorders, followed by malocclusion as the causes of bruxism (Table 2). Women, significantly more often than men chose correct answers related to the most causes of bruxism given. Whereas men significantly more often chose the answer „I don't know”, which indicated a lack of knowledge of given issue. Only, genetic factors were more often chosen by men than women, however the difference was not statistically significant..

**Table 2:** Indicating causes of bruxism by gender.

Causes of bruxism	Gender				The value of the chi^2^ test	The significance of *p*
	Male		Female			
	n	%	n	%		
Chronic stress	64	69.6	282	86.2	**13.874**	**0.0000**
Mental disorders, e.g. neurosis, depression	61	66.3	253	77.4	**4.682**	**0.0305**
Malocclusion	40	43.5	211	64.5	**11.990**	**0.0005**
Poorly fitted dentures or poorly made fillings	26	28.3	129	39.5	**3.856**	**0.0496**
Frequent chewing gum and/or biting nails	22	23.9	116	35.5	**19.716**	**0.0000**
Genetic factors	25	27.2	71	21.7	1.212	0.271
I don't know	16	17.4	18	5.5	**13.606**	**0.0002**

Women significantly more often than men participating in the survey were aware of the causes of bruxism (*p* < 0.05) (Table 3).

**Table 3:** Awareness of the causes of bruxism among genders.

Awareness of the causes of bruxism	Gender				Total	
	Male		Female			
	n	%	n	%	n	%
Yes	25	27.2	130	39.8	155	37.0
No	67	72.8	197	60.2	264	63.0
Comparison	chi^2^ = 4.876; *p* = 0.0272					

There was no significant difference in responses given by people living in city or village regarding the causes of bruxism. The only answer differentiating these two groups was “genetic factors”, significantly more often chosen by citizens than villagers (Table 4)..

**Table 4:** Indicating causes of bruxism by the place of residence of respondents.

Causes of bruxism	Place of residence				The value of the chi^2^ test	The significance of *p*
	City		Village			
	n	%	n	%		
Chronic stress	246	84.3	100	78.7	1.865	0.174
Mental disorders. e.g. neurosis, depression	221	75.7	93	73.2	0.284	0.594
Malocclusion	182	62.3	69	54.3	2.357	0.125
Poorly fitted dentures or poorly made fillings	114	39.0	41	32.3	1.734	0.188
Frequent chewing gum and/or biting nails	100	34.3	38	29.9	0.750	0.386
Genetic factors	77	26.4	19	15.0	**6.523**	**0.0106**
I don't know	19	6.5	15	11.8	3.340	0.068

The awareness of the causes of bruxism was comparable between citizens and villagers (*p* > 0.05) (Table 5).

**Table 5:** Awareness of the causes of bruxism by the place of residence of respondents.

Awareness of the causes of bruxism	Place of residence				Total	
	City		Village			
	n	%	n	%	n	%
Yes	110	37.7	45	35.4	155	37.0
No	182	62.3	82	64.6	264	63.0
Comparison	chi^2^ = 0.190; *p* = 0.663					

### 3.3 Knowledge of the effects of bruxism

Women have significantly higher general awareness of the effects of bruxism than men participating in the survey (*p* < 0.001) (Table 6)..

**Table 6:** General awareness of the effects of bruxism among genders.

General awareness of the effects of bruxism	Gender				Total	
	Male		Female			
	n	%	n	%	n	%
Yes	22	23.9	143	43.7	165	39.4
No	70	76.1	184	53.3	254	60.6
Comparison	chi^2^ = 11.813; *p* = 0.000**6**					

The general awareness of the effects of bruxism was comparable between citizens and villagers (*p* > 0.05) (Table 7).

**Table 7:** General awareness of the effects of bruxism by the place of residence of respondents.

General awareness of the effects of bruxism	Place of residence				Total	
	City		Village			
	n	%	n	%	n	%
Yes	118	40.4	47	37.0	165	39.4
No	174	59.6	80	63.0	254	60.6
Comparison	chi^2^ = 0.429; *p* = 0.512					

#### 3.3.1 Effects of bruxism on masticatory muscles and TMJ

Among the responses on the effects of bruxism affecting the masticatory muscles and TMJ, the most commonly chosen option was pain in masticatory or in facial muscles (78.0 %) (Table 8), followed by clicking in the TMJ (70.9 %). The least chosen answer was problems with opening the mouth, with only 39.6 % of respondents choosing this answer. Only 7.9 % respondents did not know the answer to the question., .

**Table 8:** Distribution of answers to the question about the effects of bruxism affecting the masticatory muscles and the TMJ (all respondents).

Effects of bruxism on masticatory muscles and TMJ	n	%
Masticatory/facial muscle pain	327	78.0
Clicking in TMJ	297	70.9
Neck pain	224	53.5
Masticatory muscle overgrowth or "square face"	202	48.2
Problems with opening the mouth	166	39.6
I don't know	33	7.9

When asked about the effects of bruxism on masticatory muscles and TMJ, women significantly more often chose all of the correct answers than men (Table 9). While men more frequently picked the statement “I don't know” to this question.

**Table 9:** Answers to the question about the effects of bruxism on the masticatory muscles and TMJ by gender.

Effects of bruxism on masticatory muscles and TMJ	Gender				The value of the chi^2^ test	The significance of *p*
	Male		Female			
	n	%	n	%		
Masticatory/facial muscle pain	58	63.0	269	82.3	**15.477**	**0.0001**
Clicking in TMJ	45	48.9	252	77.1	**27.569**	**0.0000**
Neck pain	40	43.5	184	56.3	**4.721**	**0.0298**
Masticatory muscle overgrowth or "square face"	31	33.7	171	52.3	**9.946**	**0.0016**
Problems with opening the mouth	23	25.0	143	43.7	**10.530**	**0.0012**
I don't know	19	20.7	14	4.3	**26.521**	**0.0000**

Women significantly more often than men were aware of the effects of bruxism on masticatory muscles and TMJ (*p* < 0.001) (Table 10).

**Table 10:** Awareness of the of the effects of bruxism on masticatory muscles and TMJ among genders.

Awareness of the effects of bruxism on masticatory muscles and TMJ	Gender				Total	
	Male		Female			
	n	%	n	%	n	%
Yes	26	28.3	163	49.9	189	45.1
No	163	49.9	164	50.1	230	54.9
Comparison	chi^2^ = 13.512; *p* = 0.0002					

Both, respondents residing in cities and villages, equally frequently knew the effects of bruxism on masticatory muscles and TMJ. However, village residents more frequently chose “I don't know” response to this question (Table 11)..

**Table 11:** Answers to the question about the effects of bruxism on the masticatory muscles and the TMJ by place of residence.

Effects of bruxism on masticatory muscles and TMJ	Place of residence				The value of the chi^2^ test	The significance of *p*
	City		Village			
	n	%	n	%		
Masticatory/facial muscle pain	234	80.1	93	73.2	2.465	0.116
Clicking in TMJ	215	73.6	82	64.6	3.522	0.061
Neck pain	163	55.8	61	48.0	2.265	0.132
Masticatory muscle overgrowth or "square face"	142	48.6	60	47.2	0.068	0.794
Problems with opening the mouth	124	42.5	42	33.1	3.266	0.071
I don't know	18	6.2	15	11.8	**3.889**	**0.0486**

**Table 12:** Awareness the effects of bruxism on the masticatory muscles and the TMJ by the place of residence of respondents.

Awareness of the effects of bruxism on masticatory muscles and TMJ	Place of residence				Total	
	City		Village			
	n	%	n	%	n	%
Yes	138	47.3	51	40.2	189	45.1
No	154	52.7	79	59.8	230	54.9
Comparison	chi^2^ = 1.803; *p* = 0.179					

The awareness of the effects of bruxism on the masticatory muscles and the TMJ was comparable between citizens and village residents (*p* > 0.05) (Table 12).

#### 3.3.2 Effects of bruxism on teeth and gums

When attention was focused on the effects of bruxism on teeth and gums, the greatest awareness concerned worn teeth (84.7 %) and cracking enamel or teeth (70.6 %) (Table 13). Loss of fillings, teeth mobility or gum recessions were less frequently chosen, with gum bleeding being the rarest (27.9 %). Only 6 % of respondents did not know the correct answer to this question.

**Table 13:** Distribution of answers to the question about the effects of bruxism on teeth and gums (all respondents).

Effects of bruxism on teeth and gums	n	%
Worn teeth	355	84.7
Cracking enamel/teeth	296	70.6
Loss of fillings in the teeth	206	49.2
Loosening of the teeth	179	42.7
Recessions (gums lowering)	135	32.2
Bleeding gums	117	27.9
I don't know	25	6.0

Women significantly more frequently than men chose worn teeth, tooth or enamel crack and the loss of dental fillings as a result of bruxism (Table 14). Men, on the other hand, significantly more often chose the answer “I don't know” to this question. Other effects of bruxism on teeth and gums were equally often chosen by both genders..

**Table 14:** Awareness of the effects of bruxism on teeth and gums by gender.

Effects of bruxism on teeth and gums	Gender				The value of the chi^2^ test	The significance of *p*
	Male		Female			
	n	%	n	%		
Worn teeth	68	73.9	287	87.8	**10.649**	**0.0011**
Cracking enamel/teeth	52	56.5	244	74.6	**11.338**	**0.0008**
Loss of fillings in the teeth	31	33.7	175	53.5	**11.287**	**0.0008**
Loosening of the teeth	33	35.9	146	44.7	2.261	0.133
Recessions (gums lowering)	23	25.0	112	34.3	2.814	0.093
Bleeding gums	22	23.9	95	29.1	0.942	0.332
I don't know	13	14.1	12	3.7	**14.003**	**0.0002**

Women significantly more often than men were aware of the effects of bruxism on teeth and gums (*p* < 0.05) (Table 15).

**Table 15:** Awareness of the of the effects of bruxism on teeth and gums among genders.

Awareness of the effects of bruxism on teeth and gums	Gender				Total	
	Male		Female			
	n	%	n	%	n	%
Yes	17	18.5	102	31.2	119	28.4
No	75	81.5	225	68.8	300	71.6
Comparison	**chi**^**2**^**= 5.707; *p* = 0.0169**					

There were no significant differences between city and village residents’ responses to questions on the effects of bruxism on teeth and gums (Table 16)..

**Table 16:** Awareness of the effects of bruxism on teeth and gums by place of residence.

Effects of bruxism on teeth and gums	Place of residence				The value of the chi^2^ test	The significance of *p*
	City		Village			
Worn teeth	254	87.0	101	79.5	3.805	0.051
Cracking enamel/teeth	213	73.0	83	65.4	2.459	0.117
Loss of fillings in the teeth	149	51.0	57	44.9	1.337	0.248
Loosening of the teeth	126	43.2	53	41.7	0.073	0.787
Recessions (gums lowering)	96	32.9	39	30.7	0.190	0.663
Bleeding gums	84	28.8	33	26.0	0.341	0.853
I don't know	15	5.1	10	7.9	1.182	0.277

Also, no significant difference was found between both types of residents when it comes to the awareness of the effects of bruxism on teeth and gums (*p* > 0.05) (Table 17).

**Table 17:** Awareness the effects of bruxism on teeth and gums by the place of residence of respondents.

Awareness of the effects of bruxism on teeth and gums	Place of residence				Total	
	City		Village			
	n	%	n	%	n	%
Yes	87	29.8	32	25.2	119	28.4
No	205	70.2	95	74.8	300	71.6
Comparison	chi^2^ = 0.920; *p* = 0.337					

#### 3.3.3 Other possible effects of bruxism

Most respondents who were asked about other possible effects of bruxism reported headaches (71.8 %) and sleep disturbances (67.5 %), followed by tinnitus (47.5 %) (Table 18). The least obvious effect reported by respondents was a squeak in the ears as only 30.1 % respondents chose this answer.

**Table 18:** Distribution of answers to the question about other effects of bruxism (all respondents).

Other possible effects of bruxism	n	%
Headaches	301	71.8
Problems with sleeping well	283	67.5
Tinnitus	199	47.5
Loss of prosthetic crowns	172	41.1
Squeaks in the ears	126	30.1
I don't know	32	7.6

As other effects of bruxism, most women chose headaches, whereas men pointed out sleep disturbances (Table 19). Women, significantly more often than men, chose headaches, problem with sleeping well, tinnitus and squeaks in the ears as possible effects of bruxism. Men, on the other hand, more frequent chose for the answer “I don't know” to this question..

**Table 19:** Awareness of other effects of bruxism by gender.

Other effects of bruxism	Gender				The value of the chi^2^ test	The significance of *p*
	Male		Female			
	n	%	n	%		
Headaches	49	53.3	252	77.1	**20.108**	**0.0000**
Problems with sleeping well	52	56.5	231	70.6	**6.530**	**0.0106**
Tinnitus	29	31.5	170	52.0	**12.060**	**0.0005**
Loss of prosthetic crowns	30	32.6	142	43.4	3.471	0.063
Squeaks in the ears	20	21.7	106	32.4	**3.892**	**0.0485**
I don't know	15	16.3	17	5.2	**12.554**	**0.0004**

Women were aware of the other effects of bruxism significantly more often than men (*p* < 0.001) (Table 20).

**Table 20:** Awareness of the other effects of bruxism among genders.

Awareness of the effects of bruxism on teeth and gums	Gender				Total	
	Male		Female			
	n	%	n	%	n	%
Yes	51	55.4	241	73.7	292	69.7
No	41	44.6	86	26.3	127	30.3
Comparison	chi^2^ = 11.340; *p* = 0.0008					

There were no significant differences between city and village residents in responses to the questions pertaining the other effects of bruxism (Table 21)..

**Table 21:** Awareness of other effects of bruxism by place of residence.

Other effects of bruxism	Place of residence				The value of the chi^2^ test	The significance of *p*
	City		Village			
	n	%	n	%		
Headaches	210	71.9	91	71.7	0.003	0.956
Problems with sleeping well	204	69.9	79	62.2	2.368	0.124
Tinnitus	143	49.0	56	44.1	0.845	0.358
Loss of prosthetic crowns	127	43.5	45	35.4	2.376	0.123
Squeaks in the ears	88	30.1	38	29.9	0.002	0.964
I don't know	23	7.9	9	7.1	0.078	0.780

Citizens were found to be significantly more aware of the other effects of bruxism than the village residents (*p* < 0.05) (Table 22).

**Table 22:** Awareness of the other effects of bruxism by the place of residence of respondents.

Awareness of the other effects of bruxism	Place of residence				Total	
	City		Village			
	n	%	n	%	n	%
Yes	214	73.3	78	61.4	292	69.7
No	78	26.7	49	38.6	127	30.3
Comparison	**chi**^**2**^**=5.904; p=0.0151**					

### 3.4 Knowledge of the bruxism therapies

Of the measures that can be taken after diagnosing bruxism, the most frequently chosen response was relaxation techniques (74.0 %), followed by relaxation splint (70.4 %) (Table 23). The least chosen option was orthodontic treatment (32.5 %).

**Table 23:** Answers to the question about treatment that should be taken after the diagnosis of bruxism (all respondents).

Measures after diagnosis	n	%
Relaxation techniques	310	74.0
Relaxation splint	295	70.4
Psychological/psychiatric consultation	259	61.8
Physiotherapy	219	52.3
Orthodontic appliances	136	32.5
I don't know	33	7.9

Again, women more commonly than men chose correct responses as to the possible bruxism therapies, such relaxation techniques, relaxation splints, psychological/psychiatric consultation or physiotherapy (Table 24). The use of orthodontic appliances was the only therapy equally frequently chosen by both women and men. Whereas men more often than women picked the option “I don't know”..

**Table 24:** Answers to the question about bruxism management divided by gender.

Measures after diagnosis	Gender				The value of the chi^2^ test	The significance of *p*
	Male		Female			
	n	%	n	%		
Relaxation techniques	56	60.9	254	77.7	**10.537**	**0.0012**
Relaxation splint	47	51.1	248	75.8	**21.115**	**0.0000**
Psychological/psychiatric consultation	45	48.9	214	65.4	**8.312**	**0.0039**
Physiotherapy	34	37.0	185	56.6	**6.019**	**0.0142**
Orthodontic appliances	27	29.4	109	33.3	0.005	0.944
I don't know	15	16.3	18	5.5	**11.542**	**0.0007**

Women were significantly more aware of the possible bruxism therapies than men (*p* < 0.01) (Table 25).

**Table 25:** Awareness of possible bruxism therapies among genders.

Awareness of possible bruxism therapies	Gender				Total	
	Male		Female			
	n	%	n	%	n	%
Yes	21	22.8	131	40.1	152	36.3
No	71	77.2	196	59.9	267	63.7
Comparison	chi^2^ = 9.226; *p* = 0.0023					

City residents significantly more often than village residents mentioned relaxation techniques and psychological/psychiatric consultations as measures that can be taken after the diagnosis of bruxism (Table 26)..

**Table 26:** Answers to the question about bruxism management by place of residence.

Possible bruxism therapies	Place of residence				The value of the chi^2^ test	The significance of *p*
	City		Village			
	n	%	n	%		
Relaxation techniques	228	78.1	82	64.6	**8.400**	**0.0038**
Relaxation splint	210	71.9	85	66.9	1.057	0.304
Psychological/psychiatric consultation	190	65.1	69	54.3	**4.323**	**0.0376**
Physiotherapy	151	51.7	68	53.5	0.119	0.730
Orthodontic appliances	92	31.5	44	34.7	0.398	0.528
I don't know	21	7.2	12	9.5	0.621	0.431

No significant differences were found in the awareness of possible bruxism therapies among the residents of city and village (*p* > 0.05) (Table 27).

**Table 27:** Awareness the possible bruxism therapies by the place of residence of respondents.

Awareness of possible bruxism therapies	Place of residence				Total	
	City		Village			
	n	%	n	%	n	%
Yes	111	38.0	41	32.3	152	36.3
No	181	62.0	86	67.7	267	63.7
Comparison	chi^2^ = 1.257; *p* = 0.262					

## 4 Discussion

The outcomes of the present study are based on the anonymous online survey that was carried out to assess the awareness and knowledge of bruxism, its causes, effects and possible management therapies, in student population. Studies assessing the awareness of bruxism in different populations have been carried out mainly in the form of telephone calls and direct surveys [28,29] and by means of online questionnaires (Google Forms) [30].

A pronounced awareness of bruxism in the population is associated with a tendency to over- or under-report bruxism symptoms [31]. Although the data from the literature are inconsistent and vary considerably, probably due to the differences in the studied populations and in the method of research, awareness of bruxism ranges between 6 % and 23 % [29].

A telephone survey of 130 people aged 20–29 years in Istanbul showed that the frequency of the incidence of teeth clenching was 46.8 % and of teeth grinding 19.8 % [28]. According to the results of a study carried out in the Sardinian students’ population, the occurrence of the bruxism was reported by 30.6 % of subjects [29].

An online survey (also using Google Forms) conducted on a population of 100 people in India aimed to test citizens awareness and knowledge of the problems associated with TMJ disorders related to bruxism. The questionnaire contained 14 questions concerning the causes, effects, symptoms and treatment options of bruxism [30]. Comparing the studies that were carried out in South India, to those of the Polish respondents, the similarities in the given population groups can be noted. In both surveys, the respondents chose stress as the most common cause of bruxism (Hindus – 57.0 %, Poles – 82.6 %), while the second most common response was disorders of neurological origin (Hindus – 25.0 %, Poles – 74.9 %). Similarly, in both studies, respondents indicated that facial, head and ear pain were one of the most common symptoms of bruxism. An analogy was also shown in the question of possible treatments for bruxism, with both populations preferring to choose method of muscle relaxation (Hindus – 65.0 %, Poles – 74.0 %). Differences in the studies may result from, e.g. gender distribution and cultural differences [30].

Regarding the effects of bruxism highlighted by the Polish respondents in this study, three of the most common responses can be distinguished: headache, tooth wear, and pain in the masticatory and facial muscles. These symptoms are considered by patients to be the most characteristic to the problem of bruxism, as dentists try to inform their patients about the main symptoms of bruxism, paying less attention to symptoms that are difficult for non-physicians to recognize. When presenting the treatment plan to the patient, dentists inform of all the treatment possibilities, emphasizing the need for a holistic approach, which can possibly increase patient's awareness of the available treatment options.

Comparing the results on bruxism awareness in Istanbul, where the value was 45.7 % [28] and on the Italian island of Sardinia with 30.6 % [29], the authors are able to speculate that Polish students living in cities and villages are more aware of the issue of bruxism. Among answers indicating causes of bruxism, such as “chronic stress”, reported by as many as 82.6 % of respondents, or “mental disorders, e.g. neurosis, depression”, indicated by 74.9 % of respondents. In the question about the effects of bruxism, 84.7 % of Polish students chose the answer regarding tooth wear, while 78.0 % indicated “masticatory and facial muscles pain”. These differences may result from the progress in the education about the causes and effects of bruxism by dentists, as well as of publicly available information, including the Internet. This theory seems to be confirmed by the fact that the research conducted in Sardinia, in which the awareness was 30.6 % was conducted the earliest (2003) [29], whereas the survey in Istanbul conducted in 2010 showed a value of 45.7 % in terms of the awareness of bruxism [28].

The increased awareness of Polish students shown in the study may have also resulted from the increased interest in the problem of bruxism during the COVID-19 pandemic [32], during which the survey was conducted. The general psychological tension and stress caused by the COVID-19 pandemic and social isolation resulted in an increased symptoms of bruxism and TMD in the Polish, Serbian, Mexican and Israel populations and many others [17,[33], [34], [35], [36]]. There was an association between young age, female gender, being in isolation, psychological barriers, and a higher level of stress which was related to bruxism and TMD occurrence [34,36]. It should also be mentioned that university students experience higher stress levels than the general population [19].

Due to the worsening of both bruxism and other symptoms in the oral cavity, during the COVID-19 pandemic, patients preferably decided to search for information on the Internet, which was recommended by many doctors [36] and sought advice directly from doctors through “tele dentistry” [36,37].

Another issue is that the survey was conducted in January, which is the pre-exam period in Poland, usually associated with an increased feeling of stress among students and greater health neglect (lack of sleep, imbalanced diet, less sport and a lot of caffeine). It has been studied that habitual coffee consumption is a risk factor for the increased intensity of sleep bruxism [38]. Research also shows that stress increases the occurrence of bruxism, which makes it relatively easy to monitor and use as a clinical indicator of the severity of stress [19]. Due to this phenomenon, students may have been more aware of bruxism during this period, which may have resulted in higher results in the survey.

The results of this study suggest that students residing in cities are in general equally aware of causes, effects and therapies of bruxism as those living in village areas. Therefore, our hypothesis that residents of urban areas have greater awareness of bruxism than village population can be rejected. However, when it comes to pointing out the causes of bruxism, city residents significantly more often than village residents indicated genetic factors. Whereas, village residents more often chose “I don't know” option, which indicates the lack of information, when it comes to the effects of bruxism on masticatory muscles and TMJ and they were significantly less aware of other effects of bruxism than city residents. Moreover, city residents significantly more often than village residents indicated relaxation techniques and psychological or psychiatric consultations as possible bruxism therapies. All this may indicate the need for further education of young adults residing in villages on possible effects and therapies for bruxism.

In this study, women constituted the vast majority of respondents (78.0 %). Women proved to be much more aware of the causes of bruxism compared to the group of male respondents. Women significantly more often chose specific responses, which may indicate their greater awareness. Men, on the other hand, were more likely to choose the answer “I don't know”. Only one response concerning genetic factors in bruxism was shown not to be significantly different between women and men The same applies to the effects of bruxism and possible therapeutic measures. The above conclusions confirm our hypothesis that women and city residents are more aware of the causes, effects and treatment options of bruxism.

The study conducted in the Dutch adult population showed that women more frequently than men reported awake (6.4 % vs 3.2 %) and sleep (18.6 % vs 13.9 %) bruxism [39].

In the study on the Sardinian population [29], the percentage of women participants was 53.5 %, while the awareness of bruxism was 27.2 %. The higher proportion of female gender participants in the Istanbul study (58.9 %), the higher level of awareness among this population (45.23 %) [28]. In this survey of Polish students, women accounted for 78 %, which may result in increased awareness of bruxism. As a result of the obtained values, it can be concluded that the female gender determines the increased awareness of the causes and effects of bruxism. This would be confirmed by studies conducted by the COVID-19 pandemic among non-hospitalized isolated patients in a hotel, in which was admitted that women reported facial pain more often than men [36]. It may result from closer observation of their bodies or from increased involvement in health prophylaxis [6]. In addition, due to the higher susceptibility to stress factors in women, it can be hypothesized that the problem of bruxism occurs more frequently in women, which may lead to greater awareness of the issue.

The deviations of the results obtained in the present study in comparison to the literature and studies mentioned above may be caused by many factors, including an insufficient amount of studies on similar topics conducted in previous years as well as different populations studied i.e. general population vs. university students. Another limitation of this study is the fact that the survey was send to students of various faculties however, it did not specify the type of university. Hence, we are not able to determine the number of students of medical, technical or humanities universities. Due to the lack of this data, we are unable to determine how large a percentage of this group were students of medical faculties, who could have overstated the results of the study. This information would allow for identifying areas – faculties, year of study - where more education on bruxism could be introduced. We believe that the more educated people are on various medical conditions, including dental ones, the greater the chance of faster diagnosis and taking preventative measures and starting the treatment in the entire population.

Also, as most respondents chose chronic stress as the major cause of bruxism, the information of the year of study would allow to speculate whether the students observed the bruxism symptoms within themselves as a result of increased stress. Study shows that e.g. among Polish dental students, the highest level of stress were noticed in their first year of study [40].

In addition, respondents had the choice of specific answers, which in some cases could lead to unintended bias in the selection process and could have an impact on the research results. Additionally, the group of men was relatively small, when compared to women, so the comparison between the two groups was biased. This may have resulted in an overestimation of the survey results, which is confirmed by the above-mentioned studies. Due to the fact that this study was carried out on a group of students aged 18–25, of which vast majority declared as single, it was not possible to collect sufficient data to compare the phenomenon of bruxism awareness in different marital statuses. Additionally, the distribution of respondents in individual voivodships is uneven, which may result from the number of universities in specific regions. Moreover, in the survey, the question regarding the place of residence was not specified whether it was a place of study or permanent residence. This may have led people from village areas to identify as urban citizens due to studying in the city, which could have produced a bias in the results within these two groups. Also, the questionnaire did not diversify the urban areas based on the number of inhabitants. These issues would allow for more detailed analysis of the results.

## 5 Conclusions

It can be noticed that awareness of bruxism has increased in comparison to other mentioned studies. The increased percentage of women in the studies is consistent with the higher awareness of bruxism reported by these studies. It follows that women are more aware of bruxism than men. The results of the above study should be confirmed on a different age group to confirm the greater awareness of women in relation to men. Additionally, the study suggests a greater emphasis on educating young men about bruxism. In order to obtain more reliable data, further studies should be carried out on a larger group of respondents, with even distribution of participants among country areas and variety of universities considering the above limitations. Moreover, it would be of great value to combine the results of the survey with results of medical history and clinical examination of respondents to establish any bonds between the awareness of bruxism and actual mode of behavior of the participants with bruxism.

## Funding

No funding was obtained for this study.

## Ethics approval

The study was conducted in accordance with the Declaration of Helsinki and was approved by the Research Ethics Committee of the Medical University of Lodz (RNN/111/22/KE).

## Consent to participate

Informed consent was obtained from all subjects involved in the study.

## Data availability statement

The data that supports the findings of this study is available on request from the corresponding author. The data is not publicly available due to privacy or ethical restrictions.

## CRediT authorship contribution statement

**Mateusz Gizler:** Conceptualization, Formal analysis, Investigation, Software, Writing – original draft. **Natalia Pietrzak:** Conceptualization, Data curation, Investigation, Writing – original draft. **Klara Saczuk:** Conceptualization, Methodology, Supervision, Validation, Writing – review & editing. **Monika Lukomska-Szymanska:** Resources, Validation, Writing – review & editing. **Barbara Lapinska:** Formal analysis, Project administration, Validation, Visualization, Writing – review & editing.

## Declaration of competing interest

The authors declare that they have no known competing financial interests or personal relationships that could have appeared to influence the work reported in this paper.
