# Supplementary material for: Students’ awareness of the bruxism causes, effects and therapies
Source: Heliyon. 2023 Dec 19;10(1):e23708. doi: 10.1016/j.heliyon.2023.e23708 (PMC10776920; doi:10.1016/j.heliyon.2023.e23708)
Supplement: Multimedia component 1 [file mmc1.docx]

**Survey:**

**“STUDENTS’ KNOWLEDGE AND AWARENESS ON THE CAUSES, EFFECTS AND THERAPEUTIC MEASURES OF BRUXISM”**

In questions 1 – 5, please choose only one answer. In questions 6 – 10, you can choose more than one answer.

1. **Gender**

- Woman
- Man
- Other

1. **Age (in years)**

- …..

1. **Place of residence**

- Village
- City

1. **Voivodeship**

- Lower Silesia
- Kuyavia–Pomerania
- Lublin
- Lubusz
- Lodzkie
- Lesser Poland
- Masovia
- Opolskie Voivodeship
- Subcarpathia
- Podlaskie Vovideship
- Pomerania
- Silesian
- Holy Cross Province
- Warmia–Masuria
- Greater Poland
- West Pomerania

1. **Marital status:**

- Single
- Married
- Other

1. **Indicate possible cause(s) of bruxism (multiple choice):**

- Chronic stress
- Mental disorders such as neurosis, depression
- Malocclusion
- Poorly fitted dentures or poorly made fillings
- Frequent chewing gum and/or biting nail
- Genetic factors
- I don’t know.

1. **Indicate possible effect(s) of bruxism on masticatory muscles and temporomandibular joint (multiple choice):**

- Masticatory/facial muscle pain
- Clicking in temporomandibular joint
- Masticatory muscle overgrowth or "square face"
- Problem with opening the mouth
- Neck pain
- I don’t know.

1. **Indicate possible effect(s) of bruxism on teeth and gums (multiple choice):**

- Worn teeth
- Cracking enamel/teeth
- Loss of fillings in the teeth
- Loosening of the teeth
- Recession (gums lowering)
- Bleeding gums
- I don’t know.

1. **Indicate other possible effect(s) of bruxism (multiple choice):**

- Headaches
- Problems with sleeping well
- Tinnitus
- Squeaking noises in ears
- Loss of prosthetic crowns
- I don’t know.

1. **Indicate possible measure(s) that can be taken after diagnosing bruxism (multiple choice):**

- Relaxation techniques
- Relaxation splint
- Psychological/psychiatric consultation
- Physiotherapy
- Orthodontic appliances
- I don’t know.
